# Exploring consumer experiences of pharmacist prescribing – a scoping review

**DOI:** 10.1080/20523211.2026.2733984

**Published:** 2026-09-24

**Authors:** Sheilpa Shivangani Sen, Bandana Saini, Betty Chaar

**Affiliations:** School of Pharmacy, Faculty of Medicine and Health, The University of Sydney, Sydney, Australia

**Keywords:** Pharmacist prescribing, consumer perspectives, expanded scope, pharmacy practice, trust

## Abstract

**Introduction:**

The evolving scope of practice for pharmacists now encompasses a transition from traditional dispensing roles towards an integration of expanded clinical services, which include but are not limited to vaccinations, medication reviews, and prescribing. This scoping review aimed to explore the existing literature concerning consumer perceptions of pharmacist independent prescribing (PIP) from the patients who have experienced this service firsthand.

**Methods:**

The research question was developed, and relevant search terms, databases, and inclusion/exclusion criteria were systematically identified. This review followed the PRISMA guidelines (Tricco et al., 2018) and sources were searched from 2006 to 2026. Following the screening and full-text review processes, data was presented in a table form. The insights identified from consumers were grouped into themes. All screening was independently carried out by two members of the review team.

**Results:**

A systematic screening of the literature yielded 802 records, from which 19 papers met the inclusion criteria for full-text review, focusing specifically on consumer perspectives regarding their direct experience with PIP. The synthesis of these papers consistently indicated a favourable disposition among consumers concerning both the expanded PIP role and their utilisation experiences, tempered only by a few identified concerns.

**Conclusions:**

Given that Australia is still in the initial stages of implementing PIP, there is a need to give a voice to consumers. Overall, this review highlighted that consumers were largely supportive of the PIP role, and their experiences are mostly positive with this service. There is capacity to design and implement locally relevant studies to gauge the consumer perspectives of the PIP service and to identify ways to address the challenges and facilitate integration of PIPs in primary care.

## Background

For much of modern healthcare history, prescribing has been firmly situated within the medical profession's scope of practice. The system was characterised by a linear division of labour: physicians provided diagnostic authority and therapeutic direction, while pharmacists contributed specialised expertise in sourcing, preparing, dispensing, and advising on medicines (Abramowitz, 2009).

In recent times, the global healthcare landscape has seen a shift toward a multidisciplinary framework (Abramowitz, 2009; Nissen, 2008). Driven by the pressures of an escalating chronic disease burden and systemic strain on primary care physician workforces, there has been a systematic recalibration of prescriptive authority, allowing for the integration of non-medical practitioners such as nurses, midwives, and pharmacists (Nissen, 2008). This shift has meant a redefinition of the role of the pharmacist – moving from a supportive auxiliary to an autonomous clinician integrated within the broader therapeutic decision-making process (Abramowitz, 2009; Emmerton et al., 2005; Nissen, 2008).

Prescribing is a complex process, and according to Aronson JK, effective prescribing is conducted in several steps (Aronson, 2006). Initially, the prescriber needs to correctly diagnose, then carefully evaluate the benefit-to-harm ratio to confirm that the treatment is needed and suitable. Third, the most appropriate drug, dose, and treatment plan must be chosen for the patient, and finally, clear communication must be undertaken with the patient about the medication's expected effects, possible side effects, and the monitoring plan (Aronson, 2006). In essence, prescribing is a clinical decision-making process based on evidence, patient needs and safety.

Current clinical practice categorises pharmacist prescribing authority into two broad categories: dependent and independent (Mesbahi et al., 2025; Tsuyuki & Watson, 2020) (Table 1).

**Table 1.** Comparison of pharmacist prescribing models (Mesbahi et al., 2025; Tsuyuki & Watson, 2020).

	Dependent prescribing	Independent prescribing
**Alternative expression(s)**	Supplementary or Collaborative Prescribing	Pharmacist Independent Prescriber (PIP)
**Level of Autonomy**	Requires supervision or a formal agreement	Full autonomy within their scope of practice
**Relationship with Physician**	Works under a doctor or through a Collaborative Practice Agreement (CPA)	No direct agreement or physician oversight required for specific cases
**Clinical Process**	Often follows a pre-determined protocol or diagnosis made by a doctor	Can assess the patient and provide an initial diagnosis
**Prescribing Authority**	Prescribing authority limited to the specific CPA or protocol	Includes initiation, modification and deprescribing
**Legislative/Regulatory Framework**	Enabled through state/provincial legislation or regulatory board approval Requires a written CPA or protocol with a supervising physician	Established through national or jurisdiction-wide legislation recognising pharmacists as autonomous prescribers with defined legal authority
**Scope of Practice**	Condition-specific prescribing and medication adjustments. Chronic disease management within agreed parameters	Broad prescribing authority within professional scope
**Training & Credentialing Requirements**	Completion of accredited modules Jurisdiction-specific certification Approval by medical partner or regulatory body Ongoing competency requirements	Mandatory completion of accredited prescribing programs Supervised clinical placement and ongoing competency assessments Registration as an independent prescriber

The global landscape of pharmacist prescribing is not uniform; it is a collection of diverse regulatory models. The United States pioneered the legislative framework for pharmacist prescribing, but over the years the United Kingdom (UK) has unarguably achieved the most significant and widely recognised expansion of these professional rights (Tonna et al., 2007). Within the broader European landscape, current practice remains in various stages of emergence (Garattini et al., 2021). Switzerland, Denmark, France, and Poland have established pathways for independent prescribing, whereas nations such as Ireland and the Netherlands are in the early stages of implementation and are primarily focusing on collaborative models or the management of minor ailments (Garattini et al., 2021; Vogler et al., 2012). Most other European nations remain in the pilot and evaluation stages to ensure safety and effectiveness (Figure 1).

**Figure 1.**
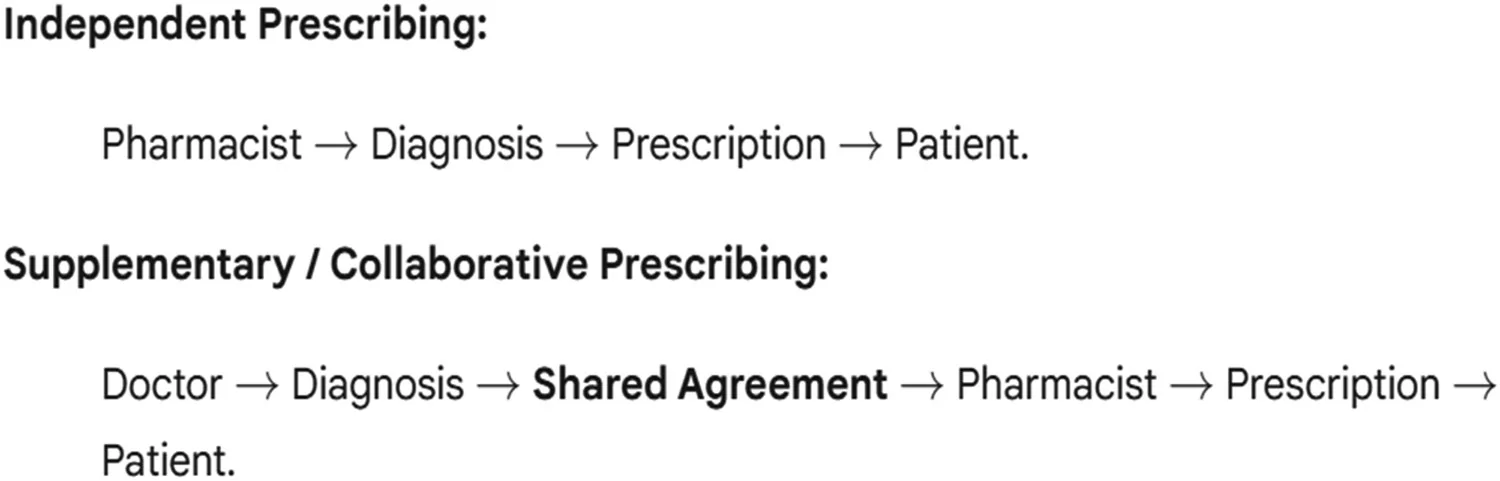
A visual comparison of workflow: independent versus dependent prescribing by pharmacists (Mesbahi et al., 2025; Tsuyuki & Watson, 2020).

In Australia, pharmacists have been prescribing according to a limited formulary for quite some time, namely ‘Pharmacy Only’ or ‘Over the Counter’ medicines (S2) and ‘Pharmacist Only’ medicines (S3) (Nissen, 2008). Australia is currently advancing initiatives to broaden the professional capabilities and prescribing authority of its pharmacy workforce; however, this revolution is largely driven by state-based pilot programs.

The state of Queensland launched its first Urinary Tract Infection Pharmacy Pilot in 2020 (Spinks et al., 2024). The successful pilot program, which allowed accredited pharmacists to prescribe antibiotics independently for uncomplicated urinary tract infections (UTIs), has since then been implemented in practice (Spinks et al., 2024) . This success spurred every Australian state and territory to implement similar pharmacist-led UTI prescribing programs, with some additionally allowing the prescribing of oral contraceptives and other minor conditions in their pilots as well (Heaney, 2024; Spinks et al., 2024).

The transition of pharmacists into high-autonomy roles, including independent prescribing, has the potential to create a misalignment between enhanced accessibility of prescribing services and patients’ established expectations of physician-centred care (Ahmed, 2025). Despite robust evidence demonstrating the safety, clinical efficacy, and economic advantages of pharmacist prescribing, Australian literature offers only a limited understanding of patient readiness for this expanded scope (Bajorek et al., 2017). In the absence of a comprehensive synthesis of patient trust, perceived professional boundaries, and psychological readiness, the rapid evolution of pharmacists’ clinical responsibilities may exceed public acceptance and threaten the long-term sustainability of these upcoming independent prescribing models (Vogler et al., 2012).

This scoping review seeks to systematically map and integrate international evidence on consumer perceptions of pharmacist independent prescribing from those that have experienced this service directly, and identify shared themes across borders – such as the value placed on accessibility – while also highlighting context-specific barriers, including concerns about continuity or fragmentation of care.

Since globally pharmacist prescribing models vary significantly, ranging from independent prescribing privileges in some jurisdictions to structured collaborative practice agreements, emergency supply provisions, and informal over-the-counter supply practices in others. These diverse regulatory frameworks and scope-of-practice limits shape the baseline role of pharmacists within local healthcare systems. Consequently, international differences in regulations and medicine access directly influence consumer expectations, trust, perceived novelty, and overall acceptance of pharmacist prescribing services.

The insights from this review will help inform policy and practice by ensuring that the progression of pharmacist independent prescribing remains aligned with patient expectations, supports informed consent, and strengthens public confidence and trust in the profession of pharmacy.

## Methods

### Scoping review

As described by Arksey and O’Malley (2005), scoping reviews aim to map the existing literature on a topic, particularly when the research area is complex or has not been extensively reviewed. They are useful for clarifying key concepts and identifying gaps in the literature (Arksey & O’Malley, 2005). This scoping review is an attempt to synthesise the literature on public attitudes towards pharmacists’ independent prescribing and will provide a foundation for future research on this topic.

### Search strategy

An extensive search of the following databases was initially conducted: Medical Literature Analysis and Retrieval System Online (MEDLINE via OVID), Excerpta Medical Database (Embase via OVID), Scopus (via OVID) and Cumulative Index to Nursing and Allied Health Literature (CINAHL via EBSCO). Appropriate search terms including both keywords and MeSH headings were identified and customised for each database. A search was conducted across titles, abstracts, and keywords using Boolean operators.

In addition to these primary database searches, grey literature was identified by searching the ProQuest Dissertations & Theses Global database, International Pharmaceutical Abstracts (IPA) database, and also by manually screening the reference lists of included studies. We also performed a targeted Google Scholar search to identify any relevant non-peer-reviewed technical reports.

The initial database searches were carried out between 1 September 2025 and 30 November 2025, and all databases were re-run on 30 May 2026 to ensure the inclusion of the most recent publications prior to finalising the review. Equivalent strategies for all the databases mentioned above and their relevant syntaxes are reported in full in Supplemental File 1.

### Inclusion and exclusion criteria

Studies were included if they were describing original research, published in English from 1 January 2006 to 30 May 2026. The year 2006 was chosen as the defining point because independent pharmacist prescribing came into effect in the UK at this time, and this timeframe generally reflects the introduction and expansion of pharmacist independent prescribing internationally.

Studies were selected only if they incorporated consumer perspectives on the prescribing activities of pharmacists. Studies were eligible if they evaluated first-hand experiences of consumers who had directly received a pharmacist independent prescribing service. Proxy reports from carers were included only where they reflected on a specific, completed consultation received by a dependent (for instance, paediatric or cognitively impaired patients). Studies assessing hypothetical willingness, prospective attitudes, or general public opinions without direct service exposure were excluded.

Review articles, editorials, opinion articles, reports, letters or free communications, book chapters, unpublished studies, or event abstracts were excluded, as well as subject matter that diverged from the topic of interest in the present review.

### Study selection

All records retrieved from databases and Google Scholar were imported into the Covidence software (Covidence, 2025) and de-duplicated. Screening proceeded in two stages; the first being title and abstract screening, where two reviewers (S.S. & B.C.) independently screened titles and abstracts against the pre-specified inclusion and exclusion criteria. Records were coded as ‘include’, ‘exclude’, or ‘uncertain’. Discrepancies were resolved through discussion, with a third reviewer (B.S.) when needed.

The next stage included full-text screening, whereby full texts of all potentially eligible studies were retrieved and once more assessed independently by two reviewers (S.S. & B.C.). Reasons for exclusion at this full-text stage were documented for each study. A PRISMA-ScR (Tricco et al., 2018) flow diagram summarises the number of records identified, screened, excluded, and included at each stage (Figure 2). Where studies reported mixed prescriber groups (for instance, nurse and pharmacist prescribers or non-medical prescribers collectively), only data explicitly relating to pharmacists’ independent prescribing roles or data clearly attributable to pharmacist independent prescribers were extracted and included in the synthesis.

**Figure 2.**
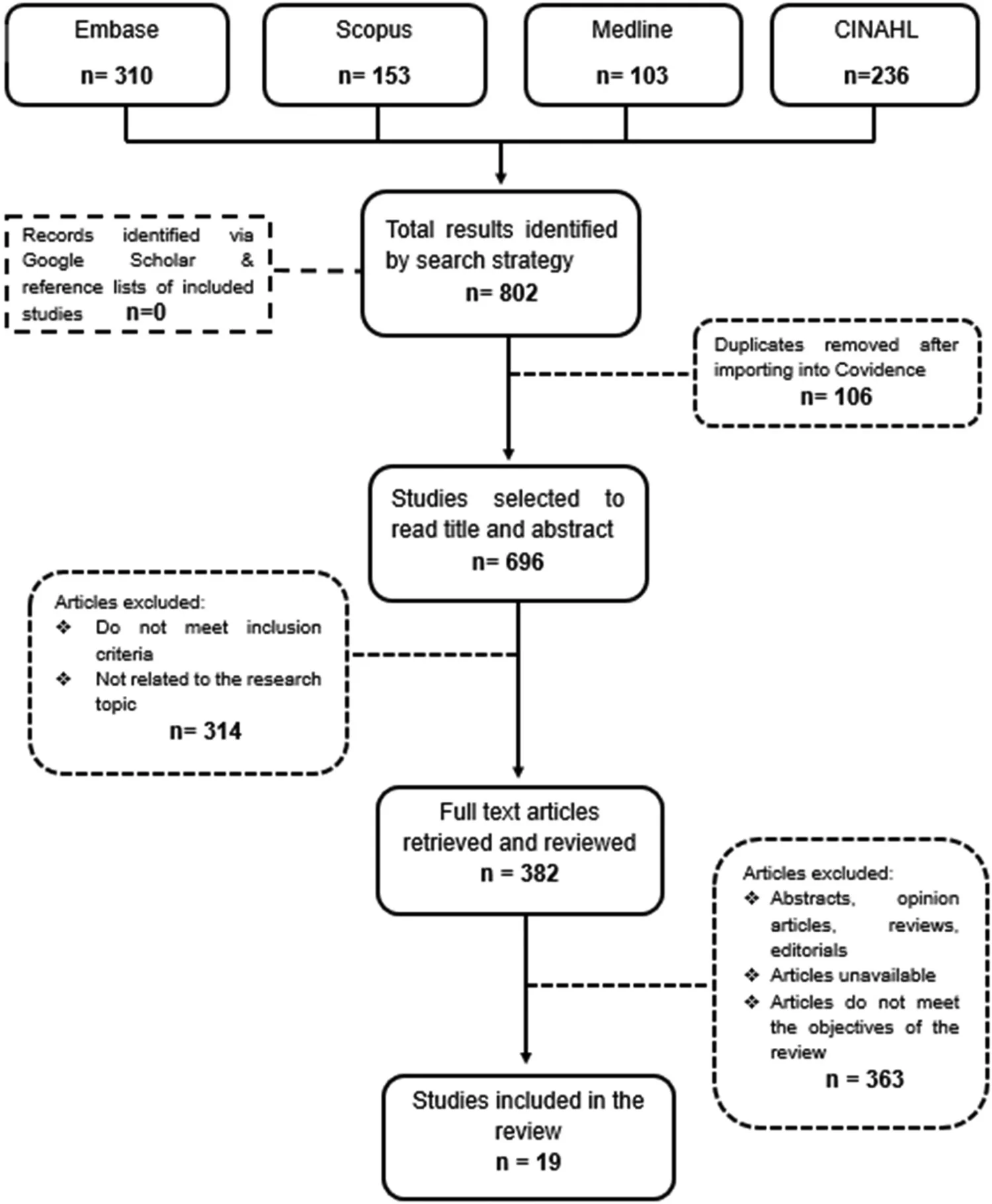
PRISMA flow diagram (search strategy and study selection).

### PRISMA-ScR reporting

We followed the Preferred Reporting Items for Systematic reviews and Meta-Analyses extension for Scoping Reviews (PRISMA-ScR) (Tricco et al., 2018) throughout the design, conduct, and reporting of this review. The completed PRISMA-ScR checklist, indicating where each item is addressed in the manuscript, is provided as Supplemental File 2.

### Data synthesis

All articles meeting the inclusion criteria underwent analysis and data extraction, with the information presented in a tabulated form. The following characteristics were recorded for each study included in the review: lead author, year, location, research objective, research design, methodology, participants, key findings and limitations (Supplemental File 3).

Where studies involved multiple prescriber groups (e.g. nurse and pharmacist prescribers, non-medical prescribers collectively), only data explicitly attributable to pharmacists’ prescribing roles were extracted. For general-practice pharmacist studies, only prescribing-related interactions (for instance, initiation, de-prescribing, clinical decision-making) were retained. Non-pharmacist data were not synthesised.

## Results

The initial database searches yielded 802 results, which were subjected to the screening process. This involved initially removing duplicates and non-research articles, followed by a review by title and abstract based on inclusion criteria. Finally, after completing full-text screening, 19 articles were identified that were suitable for inclusion in the scoping review.

Of the 19 articles included, eight were quantitative (Beahm et al., 2018; Houle et al., 2018; Mansell et al., 2015; Newlon et al., 2022; Rafie et al., 2022; Rodriguez et al., 2020; Stewart et al., 2011; Tinelli et al., 2015), seven qualitative (Al Harmarneh et al., 2018; Graham et al., 2020; Karampatakis et al., 2021; Mann et al., 2022; McCann et al., 2015; Officer et al., 2021; Speirits et al., 2021), and the rest were mixed-method studies (Alshehri et al., 2023; Hindi et al., 2019; MacDonald et al., 2023; Thomas et al., 2023). Quantitative studies utilised surveys which were either paper-based or online. The qualitative studies employed patient interviews and focus groups, whilst the mixed-method studies used a combination of the above.

The studies were geographically diverse, with five originating from Canada (Al Harmarneh et al., 2018; Beahm et al., 2018; Houle et al., 2018; MacDonald et al., 2023; Mansell et al., 2015) and five from England (Alshehri et al., 2023; Hindi et al., 2019; Karampatakis et al., 2021; Thomas et al., 2023; Tinelli et al., 2015). Additionally, three studies were conducted across the UK (Graham et al., 2020; Mann et al., 2022; Speirits et al., 2021), three from the USA (Newlon et al., 2022; Rafie et al., 2022; Rodriguez et al., 2020), one from Northern Ireland (McCann et al., 2015), one from New Zealand (Officer et al., 2021), and one spanning England and Scotland (Stewart et al., 2011).

The studies included in this scoping review primarily focused on pharmacists as independent prescribers. However, some varied studies were included, even though they did not exclusively focus on pharmacy, given the limited research directly addressing pharmacist independent prescribing on its own. One study (Stewart et al., 2011) took a unique approach by examining patient perspectives for pharmacists in both supplementary and independent roles. Other research (Officer et al., 2021; Tinelli et al., 2015) broadened the scope and investigated patient perspectives on both nurse independent prescribers (NIPs) and PIPs. Another study (Hindi et al., 2019) offered an even wider lens, exploring the views of NIPs, PIPs, prescribing podiatrists, their colleagues, and patients regarding independent prescribing rights. Additionally, these two studies (Alshehri et al., 2023; Thomas et al., 2023) considered both patient and PIP perspectives.

### Positive perceptions

The consumers generally expressed a positive outlook on both the PIP role and their direct experiences of the service (Alshehri et al., 2023; Graham et al., 2020; Mann et al., 2022; McCann et al., 2015; Rafie et al., 2022; Rodriguez et al., 2020; Speirits et al., 2021; Stewart et al., 2011; Thomas et al., 2023), as evidenced by four major positive themes identified (Table 2).

**Table 2.** Thematic analysis of consumer experiences of Pharmacist Independent Prescriber (PIP) services.

**Positive themes**
1. Impact on healthcare- the bigger picture (a) Reduce strain on healthcare (MacDonald et al., 2023) (b) PIP ideally will fill healthcare gaps (Al Harmarneh et al., 2018) (c) Ease the workload for doctors (Alshehri et al., 2023) (d) Provide a holistic service (Beahm et al., 2018;Graham et al., 2020) (e) PIP consultation was thorough (McCann et al., 2015; Stewart et al., 2011) (f) Patients were able to ask questions about their medications (Hindi et al., 2019) (g) Accessibility and convenience • High accessibility (Al Harmarneh et al., 2018; Alshehri et al., 2023; Beahm et al., 2018; Karampatakis et al., 2021; MacDonald et al., 2023; Mann et al., 2022; Newlon et al., 2022; Officer et al., 2021; Rodriguez et al., 2020) • Greater convenience (Alshehri et al., 2023; Rodriguez et al., 2020; Thomas et al., 2023) • Shorter wait times to get an appointment with a PIP (Hindi et al., 2019; Karampatakis et al., 2021;Rafie et al., 2022) • PIP was approachable (McCann et al., 2015; Stewart et al., 2011)
2. Trust (a) Pharmacist has specialist knowledge about medications which is good for prescribing duties (Alshehri et al., 2023; Graham et al., 2020; Hindi et al., 2019; MacDonald et al., 2023) (b) Trust in pharmacist (Beahm et al., 2018; Graham et al., 2020) (c) Satisfied and confident that PIP services are equal to that of a doctor (Mansell et al., 2015; Stewart et al., 2011) (d) Confidence in services (Hindi et al., 2019; Karampatakis et al., 2021; MacDonald et al., 2023; Mansell et al., 2015; Officer et al., 2021; Speirits et al., 2021; Tinelli et al., 2015) (e) Patient/consumer felt safe (Hindi et al., 2019) (f) Trust in pharmacist to handle minor ailments (Mann et al., 2022; Mansell et al., 2015) (g) Patient/consumer felt comfortable (MacDonald et al., 2023) (h) Effective communication from PIP (Graham et al., 2020,28) • Easy to talk to (McCann et al., 2015), great rapport (Al Harmarneh et al., 2018,29), encouragement given for treatment (Al Harmarneh et al., 2018), supportive (Hindi et al., 2019;MacDonald et al., 2023;Thomas et al., 2023), reassurance from the PIP (Karampatakis et al., 2021), compassionate (Al Harmarneh et al., 2018), caring (Karampatakis et al., 2021), high quality interactions between PIP and patient (Karampatakis et al., 2021), PIP was informative (Speirits et al., 2021)
3. Ethical aspects of PIP experience (a) Patient/consumer felt empowered (Graham et al., 2020) (b) Shared decision making (Graham et al., 2020)
4. Consumer acceptance – Acceptance of pharmacists as prescribers: new role (a) Satisfaction of PIP service – post consultation (Al Harmarneh et al., 2018; Alshehri et al., 2023; Beahm et al., 2018; Hindi et al., 2019; Houle et al., 2018; Mansell et al., 2015; Speirits et al., 2021; Tinelli et al., 2015) (b) Positive about multidisciplinary services (Mann et al., 2022; Speirits et al., 2021) (c) Acceptability of the PIP role (Mann et al., 2022) (d) In the future: Recommend PIP services to others (MacDonald et al., 2023; Rafie et al., 2022; Rodriguez et al., 2020; Stewart et al., 2011) (e) In the future: Use PIP services again (for themselves) (Newlon et al., 2022;Rafie et al., 2022) (f) Longer consultation lengths (when compared with doctors) (Alshehri et al., 2023; Hindi et al., 2019; Houle et al., 2018; Tinelli et al., 2015).
Negative themes
1. Concerns about the PIP specialising only in one area (McCann et al., 2015)
2. Insufficient awareness of the PIP role and services (Karampatakis et al., 2021; Mann et al., 2022; McCann et al., 2015)
3. Preference given to consulting a doctor after PIP diagnosis (for a second opinion) (McCann et al., 2015)
4. Consumers are not aware of the extra training and education PIPs need to practice (McCann et al., 2015)

#### Impact on healthcare – the bigger picture

Utilising the PIP role was viewed as a considerable promise for reshaping the healthcare system. It was projected that PIPs can help bridge existing gaps in care (Al Harmarneh et al., 2018) and reduce the pressure on current healthcare resources (MacDonald et al., 2023), particularly by easing the workload of general practitioners (Alshehri et al., 2023).

A significant strength of the PIP role was perceived as its ability to make healthcare more accessible and convenient. The improved accessibility of prescribing needs was frequently mentioned in the literature included in this scoping review (Al Harmarneh et al., 2018; Alshehri et al., 2023; Beahm et al., 2018; Karampatakis et al., 2021; MacDonald et al., 2023; Mann et al., 2022; Newlon et al., 2022; Officer et al., 2021; Rodriguez et al., 2020) and some studies reported improved patient convenience (Alshehri et al., 2023; Rodriguez et al., 2020; Thomas et al., 2023) since community pharmacies are open for longer hours.

In many of the studies, patients benefitted from quicker access to appointments with a PIP (Hindi et al., 2019; Karampatakis et al., 2021; Rafie et al., 2022). Their approachable manner was highlighted as a valued aspect of the patient experience (Mansell et al., 2015; McCann et al., 2015; Stewart et al., 2011; Tinelli et al., 2015) as well as offering a holistic approach (Beahm et al., 2018; Graham et al., 2020) to patient care during consultations.

Consultations with PIPs were notably longer when compared by the patients to those with their GPs (Alshehri et al., 2023; Hindi et al., 2019; Houle et al., 2018; Tinelli et al., 2015), allowing for more in-depth discussions. Furthermore, patients felt enabled to ask more detailed questions about their medications (Hindi et al., 2019) with the PIP.

#### Trust

A strong perception of trust was evident of the PIP role, whereby positive patient experiences were underpinned by both trust and effective communication. Patients in general expressed trust in the pharmacist's capabilities (Beahm et al., 2018; Graham et al., 2020), including their ability to address minor ailments (Mann et al., 2022; McCann et al., 2015). This translated to satisfaction and confidence that PIP services were comparable in quality to those provided by a doctor (Mansell et al., 2015; Stewart et al., 2011).

The pharmacist's expert knowledge of medications instilled confidence, especially concerning prescribing duties (Alshehri et al., 2023; Graham et al., 2020; Hindi et al., 2019; MacDonald et al., 2023), fostering a sense of comfort (MacDonald et al., 2023) and safety (Hindi et al., 2019) among patients.

This strong trust was further reinforced by the effective communication exhibited by PIPs (Graham et al., 2020; Officer et al., 2021), characterised by being easy to talk to (McCann et al., 2015), establishing great rapport (Al Harmarneh et al., 2018; Speirits et al., 2021), offering encouragement (Al Harmarneh et al., 2018) and support (Hindi et al., 2019; MacDonald et al., 2023; Thomas et al., 2023), providing reassurance (Karampatakis et al., 2021), and demonstrating care (Karampatakis et al., 2021) and compassion (Al Harmarneh et al., 2018), ultimately resulting in high-quality interactions (Karampatakis et al., 2021) between the patient and the PIP.

#### Ethical aspects of the PIP experience

The ethical dimension of the PIP experience features patient autonomy. Patients reported a heightened sense of empowerment when practitioners utilised a shared decision-making approach (Graham et al., 2020). Being provided with clear options and the autonomy to choose allowed individuals to feel more in control of their health outcomes.

#### Consumer acceptance – acceptance of pharmacists as prescribers: new role

The acceptance of pharmacists in this expanded prescribing role of a PIP was evident in several findings. High levels of satisfaction were reported following PIP consultations (Al Harmarneh et al., 2018; Beahm et al., 2018; Hindi et al., 2019; Houle et al., 2018; Mansell et al., 2015; Tinelli et al., 2015).

The PIP role itself was deemed acceptable (Mann et al., 2022), and a significant indicator of this positive reception is the public's future intentions: many would reportedly recommend PIP services to others (Rafie et al., 2022; Rodriguez et al., 2020; Stewart et al., 2011; Thomas et al., 2023) and others would personally use these services again (Newlon et al., 2022; Rafie et al., 2022).

Furthermore, there was a positive sentiment towards multidisciplinary healthcare services (Mann et al., 2022; Speirits et al., 2021) where pharmacists play a pivotal part in the team.

### Negative perceptions

Despite the positive perceptions and experiences, some concerns were raised by consumers regarding the PIP role. One concern involved the potential limitation of PIPs specialising in only one clinical area (McCann et al., 2015), which raised concerns about limited application to all health needs. This could lead to a fragmentation of care if patients still need to consult their GP or other specialists for issues outside the PIP's area of expertise.

Additionally, some individuals indicated a preference for seeking a doctor's opinion even after receiving a PIP diagnosis, suggesting a need for a second opinion (McCann et al., 2015). This could stem from the established and long-standing relationship patients have with their GPs or simply a lack of understanding about the PIP's qualifications and scope of practice. This suggested that some members of the public may not yet fully recognise or accept the PIP's diagnostic capabilities even in their specialised area, potentially negating some of the intended benefits of the PIP role, such as reducing GP workload.

Another issue identified was the insufficient public awareness of the PIP role and the services they offer (Karampatakis et al., 2021; Mann et al., 2022; McCann et al., 2015). If the public is unaware of what PIPs can do, the types of conditions they can manage, or the benefits of seeing a PIP, they are unlikely to choose this option. Without adequate public education, misconceptions about the PIP role might arise, hindering trust and acceptance. This is compounded by a lack of understanding about the additional training required for prescribing and becoming a PIP (McCann et al., 2015). If the public is not made aware that PIPs undergo specialised education and training to undertake prescribing duties and manage specific conditions, they may not perceive them as qualified or competent to fulfil this expanded role.

In summary, while the overall perception of the PIP role appears positive, these negative points underscore the importance of clear communication, addressing concerns about the scope of practice, and building public trust and awareness to ensure the successful and effective integration of PIPs into the healthcare system.

## Discussion

This scoping review provides a focused synthesis of information that addresses a key gap in the current literature, shifting the focus from provider abilities and regulations about pharmacist prescribing independently to the viewpoint of consumers who have used the service. This approach examines factors that influence service adoption, including user experience, perceived risk, trust, and service uptake.

To successfully integrate pharmacist prescribing into a healthcare system, policymakers need to know more than if the practice is safe; they need to know if patients will use the service. By highlighting information on barriers and enablers to service uptake, this review provides practical insight for public health campaigns and policy making. It also sets the stage for future research, giving a voice to consumers of the PIP services here in Australia.

This scoping review has highlighted two crucial aspects: (i) the high acceptance and demand for PIP by consumers and (ii) the imperative to consider and resolve consumer concerns.

Beyond specific PIP studies, broader literature on health literacy and patient engagement consistently demonstrates that the public's understanding of healthcare professionals’ roles directly influences their willingness to utilise services (Elvey et al., 2013,2013; Waterfield et al., 2021; Awesu et al., 2018). Naturally, the effectiveness of services provided by a pharmacist independent prescriber relies upon public understanding and acceptance (Awesu et al., 2018). If patients are unaware of what services are available or how to access them, then uptake will naturally be low.

The issues of lack of public awareness alongside limited knowledge about PIP roles and the full spectrum of services they can offer were a key concern identified. A systematic review by Awesu et al. (2018) specifically noted *‘identity recognition’* as an organisational challenge that may hamper the implementation and sustainability of pharmacist independent prescribing and that a lack of public awareness and understanding of the extended prescribing role of the pharmacist could act as a barrier to its successful adoption and implementation.

Inter-professional support for pharmacist independent prescribing is high among other healthcare professionals, especially nurses and allied health practitioners (Bettenay et al., 2019). Many nurses, who are already independent prescribers, appreciate pharmacists as valuable partners. Allied health providers, such as physiotherapists and podiatrists, see pharmacists as capable prescribers who can improve the efficiency of healthcare teams ( Bettenay et al., 2019; Graham-Clarke et al., 2018).

On the other hand, the medical profession has a more complicated and wary perspective. In literature, they often show cautious optimism and support for collaborative models, and they continually raise concerns about patient safety (Blenkinsopp et al., 2008; Hatah et al., 2012; Percival et al., 2023). There have been concerns about the ethical implications and potential conflicts of interest related to independent prescribing by pharmacists in community pharmacies. This combination of prescribing and subsequent dispensing roles raises important questions about the integrity of clinical decision-making, the dilution of the patient's best interests, the risk to patient safety, and the possibility of eroding public trust (Blenkinsopp et al., 2008; Hatah et al., 2012; Percival et al., 2023).

In Australia, medical bodies are of the view that independent prescribing by pharmacists outside of a medically led team could lead to a disjointed approach to patient management, potentially resulting in duplicated efforts, missed diagnoses, increased healthcare costs and a lack of holistic care coordination (care fragmentation) (Kennedy, 2023; Spinks et al., 2024; Tsirtsakis, 2025). The Australian Medical Association–Queensland and peak medical bodies, including the Royal Australian College of General Practitioners, have consistently opposed the expansion and permanent implementation of pharmacist independent prescribing in Queensland. While state health authorities framed the expansion as a solution to primary care accessibility, medical leadership raised significant safety, ethical, and governance concerns (Tsirtsakis, 2025). This opposition was voiced before the pilot program had been fully and independently evaluated, and more importantly, no empirical evidence was cited to substantiate these claims, instead framing the decision as politically motivated and lacking in expert consultation.

While the theoretical conflict of interest is a well-established and accepted risk in the literature (Blenkinsopp et al., 2008; Deeks et al., 2018; Kennedy, 2023; Thomas et al., 2023; Tsirtsakis, 2025), the empirical evidence of its malicious, widespread occurrence is lacking. Definitive, generalisable data demonstrating the actualisation of these conflicts in the form of malicious behavioural outcomes (e.g. systematic over-prescribing or clinically unwarranted prescribing driven by financial incentives) remains scarce in published research. This could be due to the overall difficulty in measuring subjective professional motivation or the fact that pharmacists, like other healthcare professionals, are trained in and demonstrate ethical principles and are therefore highly trusted by communities worldwide.

This discrepancy could potentially undermine the validity of the claims made by medical bodies; and if future research and evaluations of pharmacist prescribing models consistently fail to identify and report any issues by the consumers, it is suggestive that these concerns, while valid in principle, may not be manifesting as significant practical issues in the real-world implementation of these roles.

Facilitating the future of PIP requires a comprehensive strategy that addresses educational, inter-professional, policy, technological, and public awareness aspects to ensure seamless integration and widespread acceptance (Phipps et al., 2018). Policy and regulatory reform of scope and training, leveraging technology, implementing sustainable funding models, patient education, and transparently addressing potential conflicts of interest to build trust are crucial to raise awareness and foster understanding of the pharmacist's expanded role, empowering patients to utilise these valuable services (Phipps et al., 2018).

### Quality of evidence, potential biases and transferability of results

Although a formal quality appraisal is not required for scoping reviews, we acknowledge that the included studies varied considerably in methodological rigour, with many relying on small or convenience sampling, pharmacist-led recruitment, low response rates, and non-validated survey instruments. These limitations, summarised in Appendix 1, introduce potential sources of bias including social desirability bias, recruitment bias, response bias, and publication bias that may have influenced the nature and positivity of reported consumer experiences.

Moreover, several studies recruited participants directly from pharmacist independent prescribing (PIP) services, often immediately following successful consultations and frequently through pharmacist invitations, which likely under-represents dissatisfied patients, individuals who declined PIP services, or those sceptical of pharmacist prescribing. As a result, the predominance of favourable perceptions in the evidence base may reflect the views of motivated or satisfied service users and settings where PIP is well-established or positively framed, rather than the full spectrum of consumer experiences. Accordingly, these findings should be interpreted with caution.

In addition, while international consumer studies provide valuable insights into perceptions of pharmacist prescribing, their applicability to the Australian context is limited. The UK, Canadian and US health systems differ substantially from Australia in governance, funding and prescribing models. For example, the UK's NHS supports long-established independent pharmacist prescribing within a centrally coordinated system, whereas Canadian and US models vary by province or state and are shaped by mixed public–private funding. These structural differences influence consumer expectations, access pathways and trust in pharmacist-provided care.

In contrast, Australia's Medicare-funded, GP-centred primary care system, combined with emerging and contested pharmacist prescribing reforms, means Australian consumers have had limited exposure to pharmacists in prescribing roles. Consequently, international findings cannot be assumed to be directly transferable, and dedicated Australian consumer research is needed to understand local expectations, concerns and acceptability.

Finally, the substantial international variation in pharmacist prescribing regulations and scope-of-practice frameworks may limit the cross-jurisdictional comparability of our findings and influence consumer perceptions differently across regions.

## Conclusion

The findings of this scoping review have mostly highlighted positive perceptions of consumers/patients who have experienced the service of PIPs except for some concerns. With the help of this scoping review, it has come to surface that independent pharmacist prescribing is well established overseas in countries such as the UK and Canada, but Australian pharmacists (and consumers) are still trying to embrace this service. Due to this, we were unable to include any local data.

Each country has its unique healthcare model, and the results do not have transferability across borders. This is a timely call for future collaborations and for Australian researchers and organisations to design and implement locally relevant studies to gauge the consumer perspectives of the PIP service to inform the development of pharmacist independent prescribing policies and practices specific to our own healthcare system and regulatory environment.

## Author contribution statement

Betty Chaar (B.C.) conceived the original idea and to formulate a research question. Bandana Saini (B.S.) helped formulate the research question. Sheilpa Sen (S.S.) carried out the literature search. All the authors worked together to analyse and manage the data. All authors discussed the results and undertook thematic analysis of the results. S.S. took the lead in writing the manuscript with consultation, supervision and feedback from B.C. and B.S.

## Data access statement

All the authors had complete access to the study data.

## Supplementary Material

Supplementary File 2_PRISMA_ScR_Checklist.docx

Supplementary File 3_Data Extraction Table.docx

Supplementary File 1_Electronic Search Strategy.docx

## Data Availability

The data underlying this article are available in the article.
