# Supplementary material for: Exploring consumer experiences of pharmacist prescribing – a scoping review
Source: J Pharm Policy Pract. 2026 Sep 24;19(1):2733984. doi: 10.1080/20523211.2026.2733984 (PMC13618192; doi:10.1080/20523211.2026.2733984)
Supplement: Supplementary file 1 [file JPPP_A_2733984_SM7456.docx]

| **Electronic Search Strategy for MEDLINE, Embase, Scopus, CINAHL and IPA** | |
| --- | --- |
| **MEDLINE** | (pharmacist* AND prescrib*) OR community pharmac* OR pharmacy practice OR pharmacy service* OR independent prescrib* OR ((nonmedical OR non-medical OR repeat) AND prescrib*)  **AND**  perspective* OR perception* OR view* OR satisf* OR opinion* OR accept* OR attitude* OR trust* OR impact* OR read* OR thought*  **AND**  stakehold* OR patient* OR consumer* OR doctor* OR physician* OR general practitioner* OR public* OR nurse* OR policy* |
| **Embase** | (pharmacist/ AND prescribing/) OR pharmacist* AND prescrib* OR community pharmacy/ OR community pharmac* OR pharmacy practice/ OR pharmacy practice OR pharmacy service* OR independent prescrib* OR ((nonmedical OR non-medical OR repeat) AND prescrib*)  **AND**  perspective* OR perception* OR view* OR satisfaction/ OR satisf* OR opinion/ OR opinion OR accept* OR attitude/OR attitude* OR trust*  OR impact* OR thought*  **AND**  stakeholder* OR patient/ OR patient* OR consumer* OR doctor/ OR doctor* OR physician/ OR physician* OR general practitioner/ general practitioner OR public* OR nurse/ OR nurse* OR policy* |
| **Scopus** | ((pharmacist* AND prescrib*) OR ("community pharmac*") OR ("pharmacy practice") OR ("pharmacy service*") OR ("independent prescrib*") OR ((nonmedical OR non-medical OR repeat) AND prescrib*))  **AND**  ((perspective* OR perception* OR view* OR satisf* OR opinion OR accept* OR attitude* OR trust* OR impact* OR thought*))  **AND**  ((stakehold* OR patient* OR consumer* OR doctor* OR physician* OR "general practitioner" OR public* OR nurse* OR policy*)) |
| **CINAHL** | (MH "Pharmacists") AND ("Prescriptions") OR pharmacist* prescrib* OR "community pharmac*" OR "pharmacy practice" OR "pharmacy service*" OR "independent prescrib*" OR (nonmedical OR non-medical OR repeat) prescrib*  **AND**  perspective* OR perception* OR view* OR satisf* OR opinion OR accept* OR attitude* OR trust* OR impact* OR thought*  **AND**  stakehold* OR patient* OR consumer* OR doctor* OR physician* OR "general practitioner" OR public* OR nurse* OR policy* |
| **IPA** | ((pharmacist* AND prescrib*) OR "independent prescrib*" OR "pharmacist independent prescrib*" OR "pharmacist prescribing" OR "pharmacy practice" OR "pharmacy service*" OR "community pharmac*" OR ((nonmedical OR non-medical OR repeat) AND prescrib*))  **AND**  (perspective* OR perception* OR view* OR satisf* OR opinion OR accept* OR attitude* OR trust* OR impact* OR thought*)  **AND**  (stakehold* OR patient* OR consumer* OR public* OR doctor* OR physician* OR "general practitioner" OR nurse* OR policy*) |
| **Google Scholar Search and Screening**  To supplement the database searches and identify relevant grey literature, we conducted a structured search in Google Scholar using combinations of the following core terms: *pharmacist prescribing, independent prescribing, non‑medical prescribing, pharmacy practice, consumer experiences, and patient perspectives*.  Searches were sorted by relevance, and the first 200 results for each search string were screened by title and abstract. Records that appeared to meet the inclusion criteria were manually recorded and subsequently entered into the same screening workflow used for the database‑derived studies.  Grey literature sources were included if they provided sufficient information on consumer experiences of pharmacist independent prescribing. | |
