# Supplementary material for: Exploring consumer experiences of pharmacist prescribing – a scoping review
Source: J Pharm Policy Pract. 2026 Sep 24;19(1):2733984. doi: 10.1080/20523211.2026.2733984 (PMC13618192; doi:10.1080/20523211.2026.2733984)
Supplement: Supplementary file 2 [file JPPP_A_2733984_SM7457.docx]

Table 2 Data Extraction Table

| Lead Author  Year  Title  Location  DOI | Research objectives | Research Design  & Methodology | Participants | Key findings | Limitations |
| --- | --- | --- | --- | --- | --- |
| Stewart et al., 2011  Pharmacist prescribing in primary care: the views of patients across Great Britain who had experienced the service  Scotland & England  <https://doi.org/10.1111/j.2042-7174.2011.00130.x> | To gather and evaluate robust data on patient views of pharmacist prescribing in Great Britain's primary care settings. | Quantitative  Data collected via patient questionnaire sent out through the mail and analysed using descriptive statistics. | All pharmacist prescribers (supplementary and independent) were invited from the register of the Royal Pharmaceutical Society of Great Britain. Consenting pharmacists were included if they met the inclusion criteria.  Consenting pharmacists carried out patient recruitment by giving out recruitment packs to five consecutive patients who had experienced their prescribing service. | - 143 patients were recruited, of which RR= 73.4% (n=105/143) - Majority of recent PIP consultations focused on chronic disease management, with hypertension being the primary reason (n=31). Other notable reasons included asthma (n=11), diabetes (n=10), and high cholesterol (n=10). - 99% had positive views of PP - 88.5% were satisfied and confident that their pharmacist prescribed as safely as their GP - PP had been thorough 97.2%, approachable 99.0% - 87.4% would recommend a PP to other people. | - Published in 2011- might need update - No mention of the survey being validated- just based on the literature. - Potential for recruitment and response bias. - The study was limited by the lack of information necessary to compare responding and non-responding pharmacists and patients. |
| MaCann et al., 2012  A patient perspective of pharmacist prescribing: ‘crossing the specialisms-crossing the illnesses’  Northen Ireland  <https://doi.org/10.1111/hex.12008> | To investigate patient views and perceptions of the pharmacist prescribing role across three different clinical settings in NI. | Qualitative  Focus group methodology followed by thematic analysis. | The patients were drawn from the caseloads of three Pharmacist Independent Prescribers (PIPs) who worked in the distinct clinical areas of: (a) primary care management of hypertension and hypercholesterolemia, (b) secondary care management of cardiovascular and diabetes, and (c) secondary care anticoagulation. | - 34 out of a total 105 invited patients were included across seven different focus groups. - Themes  a) **Team approach to care**: multidisciplinary approach preferred especially those with complex medical conditions and multi-morbidity. i. **Patient benefits**: no major disadvantages described apart from PIP specialising in only one clinical area. Patients viewed the pharmacists positively, noting their approachable and non-judgemental demeanour, and the thorough information they shared. ii. **Pharmacist’s role**: patients were positive about PP. Greater knowledge of drugs and considered their area of expertise. iii. **Doctor’s role**: Patients wanted to see a doctor for the initial diagnosis or in cases involving a serious or acute medical condition. b) **Lack of awareness**: of the PP role and the training to become a PP. | - Due to the distances, it was not feasible to combine participants from all three locations. - Not possible to identify patients being treated in the inpatient setting. - Low response rate may imply bias - Approximately, one third of invited participants took part. - Findings may not be generalizable. |
| Mansell et al., 2015  Evaluating pharmacist prescribing for minor ailments  Canada  <https://doi.org/10.1111/ijpp.12128> | To determine whether patients prescribed for minor ailments  by a pharmacist symptomatically improve within a set time frame. | Quantitative  Online survey | Patients from the province of Saskatchewan were eligible for inclusion if they were prescribed an agent by a pharmacist for an applicable condition that included any of the following: allergic rhinitis, diaper dermatitis, cold sores, canker sores, insect bites, mild acne or thrush over a one-year recruitment period at participating pharmacies.  Parents were eligible if the patient was a child. | - 90 (25.6%) pharmacies were included from the province, and 125 responses were collected from the year-long online survey. - Results indicated that:  i. Patients were satisfied with the outcome both in clinical terms and in terms of pharmacist attributes. ii. Trust in pharmacists to handle minor ailments and convenience were the main reasons for choosing them over a doctor. iii. Satisfaction with the pharmacist and the service was strong. iv. The notion that a doctor would have been more thorough was largely rejected. | - Survey was derived from the literature and pilot tested, but not validated - Conditions were mild OTC product related. - Failure to collect feedback. - Only a small proportion of the entire province participated. - Low response rate. - Data collection form lacked in depth questions and the minor conditions under study were limited. - Selection bias as PIPs were involved in the recruitment process. |
| Tinelli et al., 2015  Survey of patients' experiences and perceptions of care provided by nurse and pharmacist independent prescribers in primary care  England  <https://doi.org/10.1111/hex.12099> | To obtain feedback from primary care patients on the impact of prescribing by NIPs and PIPs on experiences of the consultation, the patient-professional relationship, access to medicines, quality of care, choice, knowledge, patient-reported adherence and control of their condition. | Quantitative  Cross sectional postal survey {two separate versions of the survey (studying consultations with either NIPs or PIPs with specific wording relating to each)}  Frequencies and valid percentages reported. | Patients consulting with either NIPs (5) or PIPs (5) in general practice in primary care were recruited from six general practice study sites.  The common conditions being prescribed for include (but are not limited to) asthma, diabetes, and secondary prevention in coronary heart disease.  Patients were selected from the database of the PIP & NIP caseload if they had been prescribed for in the past 12 months. | - 30% response rate - 149/525 NIP survey, 145/450 PIP survey - **For PIPs the most frequent reason for the consultation were:**   - hypertension (31%)  - cholesterol (13%)  - heart problems (10%)  - asthma (8%)  general medication review (6%).   - **Perceptions and experiences**: majority of the patients were satisfied with their most recent consultation with the NMP. - **Relationship with the IP**: good relationships reported. High levels of confidence with the services and involvement in prescribing decisions. - **Comparing care-NMP vs doctor** - Quality of care: no difference stated in safety or quality of care. - Support for adherence: no difference in care between either NMP or prescribing doctor. - Control of condition: no effects noted when treatment by NIP or PIP. - Access to care: fewer than half of all respondents noted down longer appointments with NMP when compared with doctor. - Access to medicine: majority noted no differences in quicker access to getting their prescriptions between NMP and doctor. | - No existing validated questionnaire. - Low response rate as researcher access was not granted to patient contact details at several sites. - Not all demographic data was made available. - Study was focussed only on common prescribed long-term conditions by NMPs and cannot be applied to all long-term conditions. - No clinical data to back up patient experiences and claims. - Patients cannot make comparison between NIP and PIP services as they may likely have experienced only one. |
| Hamarneh et al., 2018  Pharmacist prescribing and care improves cardiovascular risk, but what do patients think? A substudy of the RxEACH study  Canada  <https://doi.org/10.1177/1715163518779092> | To identify the perceptions of patients regarding pharmacists’ identification and management of patients at high risk of cardiovascular events | Qualitative  Patient interviews.  Conventional qualitative content analysis. | A sub-population of patients from the R_x_EACH study identified by the pharmacists included in this trial.  To manage cardiovascular risk pharmacists were prescribing drugs for hypertension, diabetes, dyslipidaemia and smoking cessation. | - 14 patients out of a possible 16 took part in the study. - Three main themes were identified  a) **Patient-pharmacist relationship**: patients appreciated the rapport they had, and the compassion showed to them b) **Healthcare system characteristics**: expanded scope is ideal to fill healthcare gaps and resource shortfalls, felt reassured with pharmacist-physician collaborative interchange, appreciated the accessibility of the pharmacist and viewed them as a trusted health care professional. c) **Patient reaction**: patients were extremely satisfied with the care they received from the pharmacist and reported feeling empowered by the encouragement received. | - Very small sample size - Not generalizable - Pharmacist identified patients for the interview and thus may have introduced bias. |
| Houle et al., 2018  Clinical outcomes and satisfaction with a pharmacist-managed travel clinic in Alberta, Canada  Canada  <https://doi.org/10.1016/j.tmaid.2018.02.011> | To examine the effectiveness of a  pharmacist-managed travel health clinic in Alberta, Canada including vaccine and drug therapy recommendations provided and subsequent self-reported patient adherence to these,  health issues encountered during travel and their management, and  overall patient satisfaction with the care provided. | Quantitative  Convenience sampling.  Data collection had two components   a) Chart review b) Online survey (post travel)   Quantitative analysis performed to obtain descriptive statistics. | Patients were recruited from a specific pharmacist-managed travel clinic that provided recommendations on vaccines, prescription and non-prescription medications, precautions necessary, traffic safety, and sexually transmitted disease prevention.  Prescribed vaccinations administered on-site, whilst patients who are provided with prescriptions for oral therapies (such as anti-malarial, antibiotics for traveller’s diarrhoea) get them dispensed at their regular community pharmacy. | - 103 patients were included in the chart review with 78 (76%) completing the post travel survey. - Patient satisfaction with the services at the pharmacist led travel clinic was high (94%) - 41% identified correctly that the consultant was a pharmacist, and the remainder were either unsure or thought it was a physician or a nurse. - Patients were happy with their consultation length. | - Survey invalidated - Sample size small - Results may not be generalizable to all practising pharmacists. - Convenience sampling may introduce bias. - Unable to independently verify the accuracy of self-reported data such as adherence. |
| Beahm et al., 2018  Outcomes of Urinary Tract Infection Management by Pharmacists (RxOUTMAP): A study of pharmacist prescribing and care in patients with uncomplicated urinary tract infections in the community  Canada  <https://doi.org/10.1177/1715163518781175> | The purpose of this study was to evaluate the effectiveness and safety of, and patient satisfaction with, pharmacist assessment and management of patients with uncomplicated UTI. | Quantitative  Prospective registry trial with data registered into a web-based data management system. | Patients (>19 years) who presented to 39 specific community pharmacies in the New Brunswick province   - with symptoms suggestive of UTI without a current prescription (PIP arm) - or presented with a new prescription for the treatment of UTI from another health care provider   (Physician arm)  Pharmacists performed patient  assessments for symptoms of UTI and prescribed antibacterial therapy, modified antibacterial therapy, provided education only or referred to physician, as appropriate. | - 750 patients total included. 91.5% (686/750) were included in the PIP arm of study - 88.9% had sustained symptomatic resolution at 2 week follow up, 7.2% reported adverse effects, 96.5% patient adherence noted. - 398 (53.1%) responded to the patient satisfaction survey. - **Patients felt that the pharmacists’ assessments were thorough, and they were very satisfied with the care** - **Patients expressed a high level of trust of their pharmacist and appreciated the accessibility they had.** | - Study lacked a comparator group - Several aspects of the study relied on patients’ subjective description and recollection of symptoms - Loss to follow up-8.5% - The satisfaction survey was not validated (descriptive) |
| Hindi et al., 2019  Independent prescribing in primary care: A survey of patients', prescribers' and colleagues' perceptions and experiences  England  <https://doi.org/10.1111/hsc.12746> | To examine the experiences and views on independent prescribing in primary care from the perspectives of Independent Prescribers (nurses, pharmacists, and podiatrists), their colleagues, and their patients. | Mixed methods study  Surveys with open and closed ended questions.  Separate questionnaires were developed for patients, IPs and their colleagues.  Quantitative data was analysed using descriptive statistics and qualitative data was analysed thematically. | Three participant groups   - IPs whose training was funded by HEENW in the period 2015–2017 - Colleagues of IPs from above - Five consecutive patients from each IP following a consultation | - A total of 24 patients from nine practices, 20 IPs (5% RR) & 26 colleagues from 16 practices returned questionnaires. - IPs & colleagues results- were not relevant to this review - Patient results- quantitative - Patients' experiences of their consultations with the IP were **mostly positive** with the vast majority agreeing that they were  a) very satisfied with their visit to the IP (n = 23; 96%) b) felt safe being treated by the IP (n = 23; 96%) c) able to ask any questions about their medicines (n = 22; 92%).  - Patient results- qualitative - They were very **supportive** of and confident in their IP - Patients were pleased with their IP's consultation skills, **them feel at ease** and were very **knowledgeable about medications**. - Patients found it **easier and faster to get appointments and got more time to discuss their medications** with IPs. | - Very low response rate at 5% - Surveys were collated but not validated - IPs distributed questionnaires to their patients and colleagues which could have led to recruitment/ respondent bias |
| Graham et al., 2020  More than medication: Evaluating the role of the pharmacist independent prescriber in a community team for learning disabilities and behaviours deemed to be challenging  UK  <https://doi.org/10.1111/bld.12323> | To explore the impact of the PIP on a medication clinic for people with learning disabilities and challenging behaviours from the perspectives of healthcare professionals and carers in a UK mental health setting. | Qualitative  Individual, semi-structured interviews were carried out followed by a thematic analysis | Two participant groups at a specific UK NHS mental health Trust   - People with caring responsibilities of patients with learning disabilities and challenging behaviours: purposively sampled and recruited - Staff who worked with the PIP   *Voice of the carer, in this study is being used as the “voice” of the person being cared for.  *Drugs prescribed not specified. | - Nine participants; six were staff members & three people with caring responsibilities - **Five core themes** identified in the staff cohort and **three core themes** in the carer cohort. Staff interview themes have not been highlighted as they are not relevant to this review. - Carer results- positive interactions described - Establishing authentic and interactive dialogue with the PIP. **Effective communication was highlighted.** - **Gaining trust of the PIP's role in the management of patients**. There was respect for pharmacist's knowledge of pharmacology and systemic health issues. - **Patients felt empowered to challenge ambiguity and inconsistency in prescribing practice with the help of the PIP**. | - Results are not generalizable as study took place in one community service and was related to the role of one PIP only. - Small sample size |
| Rodriguez et al., 2020  Reasons for and experience in obtaining pharmacist prescribed contraception  USA  <https://doi.org/10.1016/j.contraception.2020.05.016> | To describe the reasons women chose to obtain contraception in a pharmacy, the type of counselling they received and their satisfaction with the care in comparison with women receiving a prescription from a clinic. | Quantitative  Cross-sectional survey.  Descriptive statistics and bivariate tests reported. | Women (aged 18–50) presenting for hormonal contraception in retail and university pharmacies across four states. | - Number of total participants is 426. Clinic was 276/426 (65%), direct pharmacy 150/426 (35%) - Top reasons for why they obtained their contraception directly from a pharmacist were all related to convenience and efficient access.   25.0% no appointment was required; 24.3% prescription was about to lapse or had lapsed & convenient location 23.6%   - Satisfaction was extremely high, participants obtaining contraception directly in the pharmacy were more likely to use the same provider again (100.0%) or refer a friend (98.0%) | - No data on women who sought a direct pharmacy prescription and were unable to obtain one. - Survey not validated - Information on the counselling pharmacists provided on methods they cannot prescribe is unknown |
| Karampatakis et al., 2021  Patients’ experiences of pharmacists in general practice: an exploratory qualitative study  England  <https://doi.org/10.1186/s12875-021-01393-0> | To explore patients’ experiences of general practice-based pharmacists in three different locations in England. | Qualitative  Face to-face, semi-structured interviews via purposive sampling approach.  Interpretive thematic data analysis. | Patients who visited a pharmacist in general practice at three specific large general practices located in West London, Surrey and Berkshire following their consultation with the PP. | - Twenty participants interviewed (n=20) - Four predominant themes:   - **awareness**: existence of general practice-based pharmacists needs to be promoted. Participants were mostly ignorant of this role.  - **accessibility**: readily available to take patients’ queries, in contrast to GPs who were much more difficult to contact. Less overall waiting time also mentioned.  - **interactions**: high quality interactions, caring attitude of pharmacist; all participants left consultations feeling confident, reassured and with peace of mind.  - **feedback**: doubts about patient feedback being taken seriously. | - Limitations of ethnicity and age in the sample - Findings primarily apply to the UK - Location of the interviews may have introduced bias - Bias during data categorisation may exist |
| Officer et al., 2021  ‘She taught me’: factors consumers find important in nurse practitioner and pharmacist prescriber services  New Zealand  <https://doi.org/10.1186/s12960-021-00587-y> | To explore factors underlying consumer satisfaction with primary health care nurse practitioner and pharmacist prescriber services. | Qualitative  Semi-structured interviews.  Data analysed following the realist evaluation analysis processes. | Advanced practitioners (NPs & PPs) blindly recruited participants from a list of consumers based on a set of criteria for participant selection.  *Drugs prescribed not specified. | - Consumers of PP (n=9); NP (n=12) - Participants described a mechanism of confidence in their advanced practitioner, and this came from three main contexts. - **Being known**: needs of consumers met holistically. Approach to care is patient-centred, accessible and reflects individual needs. - **Doctors tell, advanced practitioners discuss**: increased consumer understanding of their disease states and care being provided, easily able to communicate with the NP/PP. - **Provider passion**: commitment and desire to help others. | - Modest number of participants - One interviewee participated in a very short interview when compared to others. - Current theory represents one stage of theory refinement. - Additional investigation required for delivery of patient centred care by NPs/PPs. |
| Speirits et al., 2021  Patient experiences of pharmacist independent prescriber-led post-myocardial infarction left ventricular systolic dysfunction clinics  UK  <https://doi.org/10.1111/ijpp.12662> | To explore qualitatively patient experiences of attending PIP led post-MI LVSD clinics | Qualitative  One on one semi structured interviews using sequential sampling of participants.  Thematic analysis of data was undertaken. | Suitable patients were recruited by pharmacists via sequential sampling from three specific PIP led clinics at point of discharge from three hospitals offering the post-MI LVSD clinic.  *Drugs prescribed not specified. | - 12/17 (70.6%) patients discharged from the clinic were included. - Thematic analysis resulted in six themes: - **Multidisciplinary working**: interprofessional services were synergistic and augmented each other. - **Satisfaction**: pharmacist reviews were valuable, and participants spoke positively about the experience. - **Confidence in the pharmacist**: reduced stress due to PIPs positive demeanour and detailed explanations. Great rapport. - **Comparative care**: PIP led clinic was more informative. - **Prescribing behaviours**: changes to drug therapy were incremental and unhurried. Any adverse effects were resolved at the point of care. - **Monitoring**: regular review and physiological and biochemical monitoring provided reassurance to the patients. | - Small sample size - Only a regional Scottish health authority covered, other UK countries are not covered. - Bias introduced due to pharmacist led recruitment process. - Impartiality is not guaranteed as researcher was a pharmacist. |
| Mann et al., 2022  Perspectives of pharmacists in general practice from qualitative focus groups with patients during a pilot study  UK  <https://doi.org/10.3399/bjgpo.2021.0112> | To answer the research question: what is the patient perspective of pharmacists in patient- facing general practice in the UK? | Qualitative  Focus group methodology with purposive and opportunistic sampling approaches.  Thematic analysis carried out. | Patients at a general practice where a new pharmacist role in a GP practice setting was being trialled. | - 34 patients were included across five focus group interviews. - Themes identified - **Access, awareness, and acceptability**: 31/34 had prior PIP consultation experiences and acceptability of the role was high. - **What pharmacists in general practice do**: positive outcomes with PIP role. - **How they do it**: holistic service and patients reported they trusted the pharmacist. - **The pharmacist in the GP multidisciplinary team**: patients appreciated the benefits of teamwork. - **Pharmacists in the community pharmacy**: confusion around the fact that a pharmacist would consult at the GP practice | - Study was undertaken during the pilot phase of the role, significant time has passed, over which the role has changed and developed - Most patients were aged >60 years and from a White ethnic group - Focus group methodology: one group was small (n=3), at two sites practice managers insisted on attending the group discussions. |
| Newlon et al., 2022  Impact of pharmacist prescribing through direct pharmacy access policies on access to hormonal contraception  USA  <https://doi.org/10.1016/j.japh.2021.08.010> | The objective of this study was to assess the impact of direct pharmacy access policies on women’s access to hormonal contraceptives. | Quantitative  Online survey administered by an independent party using convenience sampling.  Multiple analysis used such as descriptive, chi-squared tests, Kruskal- Wallis tests, Bonferroni corrections. | Women aged 18-44 years who have lived in Indiana or 1 of the 11 DPA states or Washington, D.C., for at least 3 months and in the past year have been interested in accessing hormonal contraceptives. | - 316 eligible responses (no response rate) - Most (78.9%) of the women in the DPA user group **agreed or strongly agreed that DPA made it easier to obtain hormonal contraceptives.** - Participants were somewhat to **extremely interested in using DPA in the future** (82.8% DPA nonuser and 80.0% Indiana group). - Women who had never used DPA felt that DPA would make it **easier to access hormonal contraceptives** (81.7% DPA nonuser and 87.7% Indiana group). | - Cross-sectional nature of the research limits establishment of a causal relationship between the implementation of a DPA policy and women’s ac cess to hormonal contraceptives. - Survey not validated - Variation in the nature and times in the implementation of DPA policies in all the different states. |
| Rafie et al., 2022  Patient experiences with pharmacist prescribed hormonal contraception in California independent and chain pharmacies  USA  <https://doi.org/10.1016/j.japh.2021.11.002> | To describe patient experiences accessing contraception via pharmacist prescribing of hormonal contraceptives in California. | Quantitative  Online survey using a cross-sectional convenience sampling technique.  Descriptive statistics were used for data analysis. | Patients were recruited by pharmacists from the 44 community pharmacies in California included in the study to get a prescription for a contraception method. | - 160 responses included in the survey (no response rate) - Main reason for accessing hormonal contraception from a pharmacy included this option being **faster than seeing a doctor** (74%) - **97% were satisfied/very satisfied with the service overall**, 96% were likely to return and 95% were keen to recommend this service to a friend. | - Recruitment was limited to 44 pharmacies only - New service thus limited patient volume for data collection. - Recruitment may not have offered to the entire eligible study population due to circumstances. - Survey was not validated |
| MacDonald et al., 2023  Target users’ acceptance of a pharmacist-led prescribing service for pre-exposure prophylaxis (PrEP) for human immunodeficiency virus (HIV)  Canada  <https://doi.org/10.1177/17151635231177027> | To determine target users’ acceptance of a PrEP-prescribing service by pharmacists in Nova scotia | Mixed methods (Triangulation)  Anonymous, self-administered online survey followed by semi-structured qualitative interviews  Snowball sampling for survey and convenience sampling for interview.  Separate qualitative and quantitative data analysis.  Survey data was analysed descriptively and interviews decoded according to 7 constructs of the TFA. | Participants were > 18 years and those eligible for PrEP in Nova scotia (MSM, TGW, persons who inject drugs and HIV-negative individuals in Sero discordant relationships). | - 148 valid survey response (no response rate). - 61.8% had used a PP service previously for any indication. - 95.8% identified as MSM or TGW - Approx 40% were current or previous PrEP users. - Themes mapped to TFA - **Affective attitude**: 92.6% of participant are comfortable with pharmacist prescribing PrEP - **Burden**: most participants felt they had access to pharmacists and 63.5% did not feel stigma - **Ethicality**: 95.3% felt that PP of PrEP would benefit the community. - **Intervention coherence**: 75% aware that pharmacists play an important role for patients taking PrEP. - **Opportunity cost**: 91.1% disagreed that PP of PrEP would damage relationships with other healthcare providers and said this would reduce strain on the health care system. - **Perceived effectiveness**: 49.3% agreed that pharmacists are knowledgeable about PrEP. Highly supportive and say that pharmacists are capable. - **Self-efficacy**: participants were confident and comfortable obtaining PrEP from a pharmacist based on previous positive experiences. | - Study was limited to just one province in Canada, and most participants were located near the city area thus non-generalisable. - Most respondents were MSM or TGW, so the third eligible patient group was underrepresented. - Possible skewed results in favour of PrEP accessing community. - Survey was not validated |
| Thomas et al., 2023  Pharmacist prescriber smoking cessation intervention during the COVID-19 pandemic  England  <https://doi.org/10.18332/tid/170580> | To evaluate the accessibility, feasibility and acceptability of a remote pharmacist prescriber smoking cessation intervention. | Mixed methods  Cross sectional study  Consumer survey and pharmacist and consumer semi-structured interviews.  Descriptive statistics were reported; themes were identified for qualitative data. | Two participant groups   - PIPs who provided smoking cessation services via remote consultations (prescribe varenicline) in Kent England were recruited for telephone or online interviews - The above pharmacists identified clients who received smoking cessation support for an online survey followed by subset of clients for a telephone interview. | - 7 pharmacists and 85 clients participated. A further 11 (out of 85) clients took part in the interview that followed.   *Pharmacist related results are not discussed as they are not relevant to this review.   - Varenicline was not received by only 3 of the clients and quitting smoking was reported by 50 (59%) of the clients by the end of the 12-week program. - **95% were positive about the service**, majority would recommend the service to others, **convenience of the service and level of support was greatly appreciated** by clients. - Themes - **Relationships**: 9/11 were impressed or grateful for the support received. - **Access to varenicline**: many clients believed that timely access to medication was vital for their successful outcomes. - **Ease and convenience of service**: remote consultations were mentioned as the best aspect especially at the time of the pandemic. | - Participants not asked whether they had external motivation for quitting, for instance, having COVID-19 - Small study size - Impact of varenicline on the success of this service cannot be separated from other components such as counselling. |
| Alshehri et al., 2023  Integration of pharmacist independent prescribers into general practice: a mixed-methods study of pharmacists’ and patients’ views  England  <https://doi.org/10.1186/s40545-023-00520-9> | To explore the integration of PIPs into GP practice and patients’ satisfaction with consultations provided by PIPs within GP practices in England. | Mixed methods  Semi-structured interviews with PIPs and a questionnaire-based service evaluation of patients who had consultations with the interviewed PIPs.  Qualitative data from semi-structured interviews and open-ended questions in the survey were inductively analysed using thematic analysis | Two participant groups  -PIPs: convenience sampling, PIPs working in GP clinic recruited  -patients: PIPs who participated in the interviews were invited to distribute the questionnaires and participant information sheets to their patients following their consultations. | - Thirteen PIPs participated in the interviews. - 77 patients who received consultations at seven different GP practices by PIPs. - Patients reported very **high levels of satisfaction with all aspects of their appointments with PIPs** - Majority of patients were **confident** with the PIP’s recommendations (97%, n = 75) and **did not see the need to further consult a general practitioner after their appointment with the PIP** (75%, n = 58). - **Identified benefits of service by PIPs include accessibility and convenience, specialist expertise, longer consultation/appointments, attention and/or helpfulness, relieving pressure of the GP workload** | - Questionnaire sample size was relatively small - PIPs distributed the questionnaire to their patients, which could lead to recruitment or response bias. - Convenience sampling may have potential for self-reporting bias, social desirability bias and recall bias as well as the researchers’ bias in the collection, analysis and interpretation of the data. |
|  |  |  |  |  |  |

NIP-nurse independent prescriber; PIP- pharmacist independent prescriber; NMP- nonmedical prescriber; IP- independent prescriber; HEENW- Health Education England North West; NHS- National Health Service; NP- nurse prescriber; PP- pharmacist prescriber; MI- myocardial infarction; LVSD- left ventricular systolic dysfunction; DPA- direct pharmacy access; TFA- Theoretical Framework of Acceptability; MSM- Men who have sex with men; TGW-transgender women
